# Case Report: Recurrent pericardial tamponade in a child with COVID-19

**DOI:** 10.3389/fped.2022.1026349

**Published:** 2022-10-24

**Authors:** Ádám Győri, Tamás Decsi, József Stankovics, Zoltán Nyul, Mária Környei, György Masszi, Evelin Leibinger, Bernadett Mosdósi

**Affiliations:** Department of Paediatrics, Clinical Centre, Faculty of Medicine, University of Pécs, Pécs, Hungary

**Keywords:** cardiac tamponade, COVID-19 in children, interleukin-1 antagonist, haemorrhagic pericardial effusion, anakinra

## Abstract

**Background:**

Pericarditis is rare in Coronavirus disease 2019 (Covid-19) infection and only a few cases were reported in children.

**Case presentation:**

We present the case of a 15-year-old boy with symptoms of high fever and worsening chest pain during COVID-19 infection. Chest computer tomography (CT) and echocardiography confirmed pericardial tamponade requiring urgent drainage. Despite antiviral drug treatment, after 18 days severe attack developed requiring repeated pericardiocentesis. High dose ibuprofen, colchicin and the interleukin-1 antagonist, anakinra were given. Clinical symptoms and laboratory parameters improved after seven days of treatment. Autoinflammatory diseases were also suspected in the background the severe pericarditis, but genetic analysis ruled out any mutations.

**Conclusion:**

Pericarditis associated with COVID-19 infection may present in the acute phase or later as MIS-C. Though pericardial tamponade related to ongoing Covid-19 infection is rare in children, even biological treatment with interleukin-1 antagonist may be needed to control the inflammation.

## Introduction

After more than two years of the Covid-19 pandemic, with more than 515 million cases and more than 6 million deaths worldwide, it can be concluded that children are usually less severely affected by Covid-19 infection than adults. One-third of pediatric cases are asymptomatic, whereas in symptomatic children fever is the most common sign, followed by rhinorrhea, cough and sore throat, but diarrhea, vomiting, headache, fatigue, myalgia, tachypnea, tachycardia and rash can also occur (1). Critically severe conditions rarely develop during COVID-19 infection (2).

However, 4–6 weeks after COVID-19 infection, children may develop severe inflammation affecting multiple organs, known as Multisystem Inflammatory Syndrome in Children (MIS-C). It has multisystem involvement with fever, mucocutaneous and gastrointestinal symptoms, but the cardiovascular manifestations such as cardiac dysfunction, decompensation and shock are the most prominent. Elevated plasma concentrations of C-reactive protein (CRP), interleukin-6 (IL-6), ferritin, D-dimer and fibrinogen can be detected and can be used to establish the diagnosis (3, 4). The rapid antigen test is usually negative in MIS-C, positive serological tests, antinucleocapsid and antispike antibodies indicate previous infection (5).

## Case presentation

A previously healthy, 15-year-old boy was admitted to our department with a two-day history of high-grade (e.g., 39.2 °C) fever, worsening chest pain and dyspnoea. Both family and personal medical history was unremarkable, except for a cholecystectomy at the age of 14 years.

**Figure 1:**
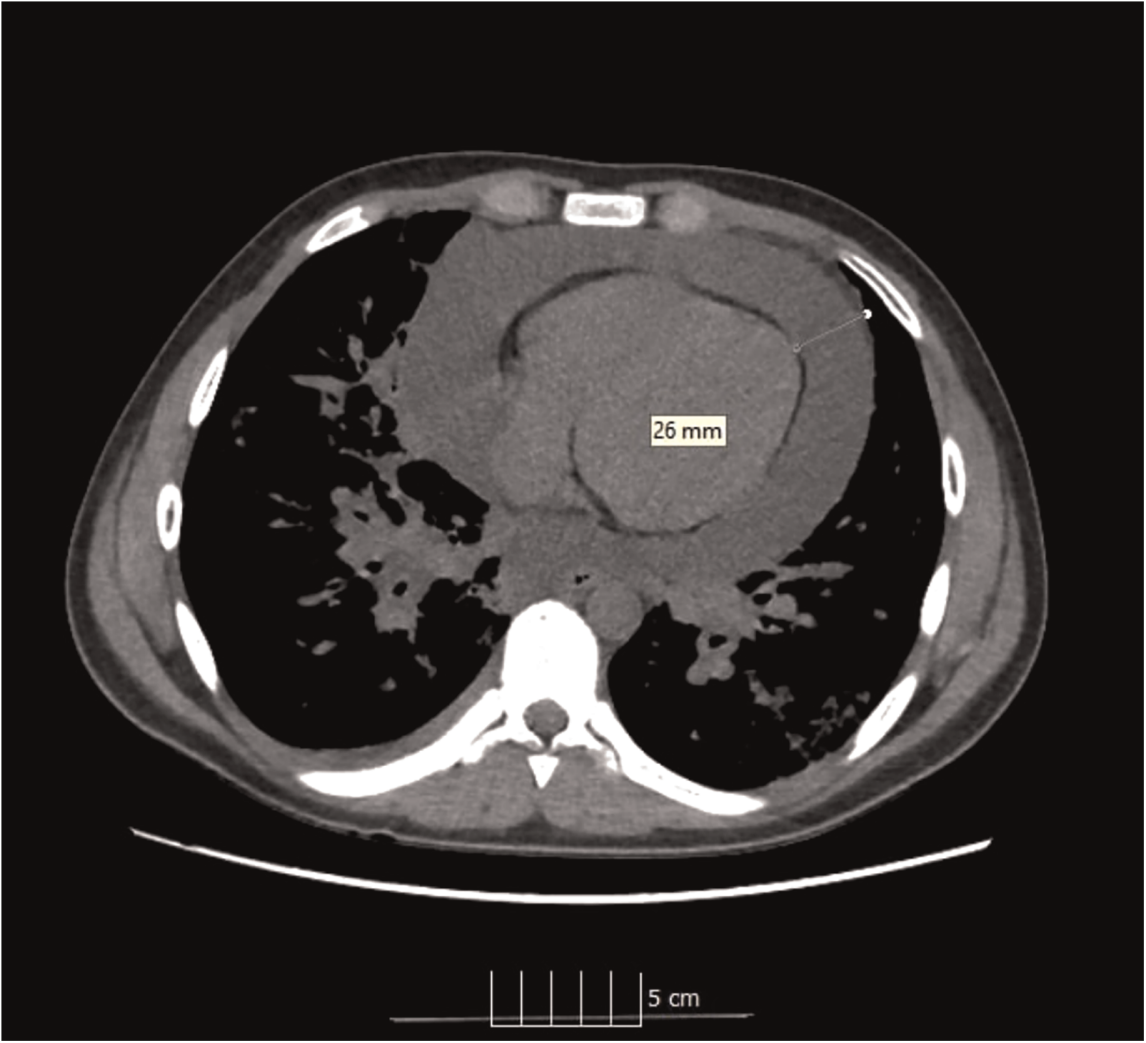
Ct image of pericardial tamponade.

**Figure 2:**
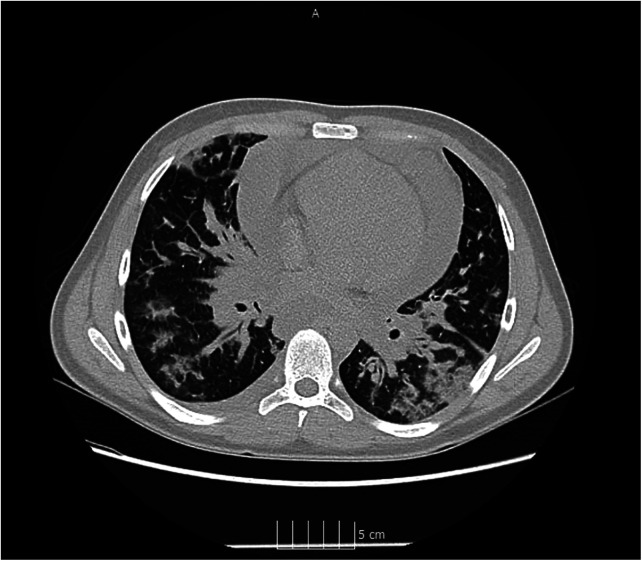
Ct image of COVID-19 pneumonia.

Physical examination revealed high temperature (39.1 °C), tachycardia (125 bpm) and dyspnea requiring immediate oxygen therapy (peripheral oxygen saturation on room air was 85%). Mild hypotension (Hgmm 90/55 mmHg) was detected. Radial artery was palpable but weak. The patient was hemodynamically stable at this stage of the disease. Covid-19 antigen test and reverse transcription-polymerase chain reaction (PCR) of nasopharyngeal swab were positive. Our patient has not been vaccinated against the virus. COVID-19 infection was also confirmed in the parents by a rapid test. A chest CT scan was performed in view of the severe respiratory symptoms and COVID-19 positivity. Multifocal, bilaterally thickened interlobular and intralobular lines in combination with a ground glass pattern was detected, the CT Covid-19 score was 16/25. A 26 mm wide circumferential pericardial effusion was identified around the ventricles (Figures 1, 2). Electrocardiogram detected low QRS voltage, but ST-elevation or other electrical alteration was not observed. Characteristic echocardiographic abnormalities for pericardial tamponade, such as right atrial diastolic collapse and paradoxical septal motion, were seen.

Laboratory tests showed elevated C-reactive protein (65.8 mg/L), ferritin (394 µg/L), interleukin-6 (116.4 pg/ml), D-dimer (3,035 µg/L) concentrations with leukocytopenia (4110/µl) and lymphocytopenia (780/µl). Procalcitonin (PCT), pro-brain natriuretic peptide (BNP) and troponin T concentrations were within the normal range (Table 1). To rule out other aetiology of pericarditis, infectious serology test (Epstein-Barr virus, cytomegalovirus, varicella-zoster virus, human immunodeficiency virus 1–2, parvovirus B19, hepatitis -A, -B, -C, -E viruses, enterovirus, Chlamydia pneumoniae, Mycoplasma pneumoniae) and autoimmune serology test (anti-nuclear, anti-nucleosome, anti-dsDNA, anti-centromere, anti-C1Q, extractable nuclear, anti-saccharomyces cerevisiae, anti-neutrophil cytoplasmic, anticardiolipin, anti-beta-2- glycoprotein, antiprothrombin antibody) were done with negative results.

**Table 1:** Laboratory markers of COVID-19 related pericarditis in our case.

	At first admission	Before anakinra treatment	After anakinra treatment
White blood cell number (/µl)	4110	10,080	9350
Lymphocyte number (/µl)	780	930	1050
CRP (mg/L)	65.8	7.4	0
Procalcitonin (ng/ml)	0.75	0.05	0.08
D-dimer (µg/L)	3035	4493	1735
IL-6 (pg/ml)	116.4	18	1.5

Emergency pericardial drainage was performed; the haemorrhagic pericardial fluid was 1200 ml in volume. Laboratory results showed the fluid to be transudate. Pathology analysis ruled out malignancy, acute inflammation was reported. Bacterial culture was negative. Medication like favipiravir (1. day: 2 × 1,600 mg, after than 2 × 600 mg for 4 days), remdesivir (1. day: 1 × 200 mg, after than 1 × 100 mg for 4 days), dexamethasone (1 × 6 mg), enoxaparin (1 × 8,000 NE) and acetyl-salicylic acid (1 × 100 mg) has been introduced. His symptoms improved significantly, and the pericardial drain was removed after 48 h. Laboratory parameters were normalized within six days. Echocardiography control showed only minimal periventricular effusion, on CT scan some regression of the Covid-19 pneumonia was detected (CTSI: 12/25). The patient was discharged after and remained on dexamethasone, enoxaparin and acetyl-salicylic acid treatment.

Four days later, he was readmitted with sudden onset of sharp chest pain and mild hypotension (blood pressure: 92/70 mmHg). Pulmonary embolism was excluded by angio-CT, but pericardial tamponade (14–16 mm wide) and progression of Covid-19 pneumonia (CTSI: 18/25) were both identified. During urgent pericardial drainage, 500 ml of sanguinolent fluid can be drained. A ClotPro® test was performed to rule out coagulation abnormalities underlying the recurrent haemorrhagic tamponade, which was negative. High-dose ibuprofen (3 × 600 mg/day) and colchicine (3 × 0.5 mg/day) as well as the IL-1 antagonist, anakinra (1 × 100 mg/day) were introduced.

After 7 days of biological treatment, echocardiography showed normal heart function, pulmonary function tests revealed mild hyperinflation with normal diffusion capacity. The therapy was well tolerated without any side effect. During a 12-month-long follow-up period, the patient remained asymptomatic and no cardiological abnormality was observed. Serological tests after the second clinical admission showed low antibody titres, suggesting a recent infection (Table 2). Since recurrent pericarditis could be a major sign of autoinflammatory disease (AID) (6), genetic tests were performed, but no mutations were found in the genes coding familial mediterranean fever, tumor necrosis factor receptor-1, mevaloante-kinase, Nod-like receptor caspase 4, NLR family pyrin domain containing 1, 3 and 12, phospholipase C gamma 2 and proteasome 20S subunit beta 8.

**Table 2:** COVID-19 antibody titres of the patient.

	At second admission	3 months after the infection	4 months after the infection	6 months after the infection	9 months after the infection and two dose of Pfizer-Biontech vaccine
Anti-covid-nucleocapsid (U/ml)	37.1	134	150	110	87.3
Anti-covid-spike (U/ml)	707	2,045	2,585	3,400	>12,500

## Discussion

To the best of our knowledge, this is the first case report of a pediatric patient with recurrent pericardial tamponade related to Covid-19 infection (7, 8).

Pericarditis is an inflammatory condition of the pericardium. It can be of infectious (viral, bacterial, fungal) or non-infectious (autoimmune, metabolic, neoplastic, iatrogenic, traumatic or drug-related). While viral (caused mainly by enteroviruses, herpesviruses and parvovirus B19) (9), or idiopathic pericarditis are usually not severe, bacterial pericarditis (caused by Mycobacterium tuberculosis, Coxiella burnetii, Borrelia burgdorferi, Pneumococcus spp., Meningococcus spp., Gonococcus spp, Streptococcus spp and Staphylococcus spp.) can be life-threatening. In our case, serological tests ruled out both viral and bacterial aetiology. Pericarditis can also be a clinical sign of autoimmune disease such as systemic lupus erythematosus, systemic sclerosis, Kawasaki-disease or AID. Immunoserological and genetic studies have also excluded these disorders (10, 11). Hemorrhagic pericarditis can develop as a complication of chest trauma, heart surgery, tuberculosis or direct neoplastic invasion, but none of these possibilities can explain the sanguinolent fluid in our patient (12). Extrapulmonary complication caused by COVID-19 is rare but can be serious. Cardiac dysfunction occur in less than 5% of hospitalized patient and often coexist with pulmonary involvement. Although 10% of adults develop severe cardiovascular involvement during Covid-19 infection, cardiac dysfunction such as myocarditis, arrhythmias, myocardial injury and cardiomyopathy are rarely seen in pediatric cases (13–15). It should be emphasised that severe cardiac involvement in children with COVID-19 infection is mainly part of MIS-C. The clinical features of severe acute COVID-19 and MIS-C overlap. Organ system involvement and laboratory abnormalities may help differentiate severe acute COVID-19 from MIS-C. Severe pulmonary involvement, which is a prominent feature of acute infection, in MIS-C is usually secondary to impaired myocardial function or shock. Inflammatory markers, antiviral antibody titres and cardiac enzymes are more elevated in MIS-C than in acute COVID-19 (16). Our patient had severe pulmonary involvement characteristic of COVID-19 infection and laboratory findings such as rapid antigen and PCR test positivity, just mildly elevated inflammatory marker confirmed acute infection. Cardiac enzyme abnormalities characteristic of MIS-C were not detectedable in our patient. Given that our patient had severe respiratory symptoms during Covid-19 infection, the first diagnostic procedure was CT scan followed by complex cardiological investigations. Typical signs of tamponade were seen on CT, ECG and echocardiography (17–19). Cardiac tamponade rarely occurs in children. It is usually related to cardiac surgery, neoplasia, uremia, infection, autoimmune or AID syndromes, and 5% of cases are idiopathic. The prognosis depends on prompt recognition of the condition and management of the underlying cause.

Only just four cases of Covid-19-associated pericarditis have been reported in the literature (20–22). Both similarities and differences were found between the individual cases. Worsening chest pain was the leading symptom in all cases, fever was not always associated. Apart from our case, only one case of pulmonary involvement was detected. Cardiac involvement is most often associated with pulmonary involvement, therefore, in case of chest pain, a cardiological examination is necessary in case of chest pain with a negative chest x-ray. Electrocardiography showed abnormalities in only 2 cases, and the diagnosis was made by echocardiography. Only one child has been reported in the literature to have cardiac tamponade. None of the laboratory abnormalities characteristic of MIS-C, suggestive of myocardial involvement, were detected. Laboratory alternations most consistent with COVID-19 were elevated CRP, elevated erythrocyte sedimentation rate and lymphocytopenia. The severity of lymphocytopenia reflects the severity of COVID-19, which was only observed in our case. All children were treated with high doses of ibuprofen, and only in our own patient, due to recurrent tamponade, IL-1 antagonist treatment was introduced. To date in the literature, one child becased our patient has undergone pericardiocentesis for severe pericarditis associated with COVID-19 infection.

Exact pathomechanism is unknown. Severe clinical signs may be observed under the age of one year or in the presence of comorbidities such as obesity, asthma, diabetes mellitus, cancer. None of these were present in our patient. There is no protocol for the treatment of pediatric pericarditis associated with Covid-19. First line treatment with high-doses of aspirin, non-steroidal anti-inflammatory drugs and colchicine may be effective in pericarditis, but in recurrent cases such as ours, biological treatment with IL-1 antagonist may be necessary to control inflammation (23–25).

## Conclusion

**Figure 3:**
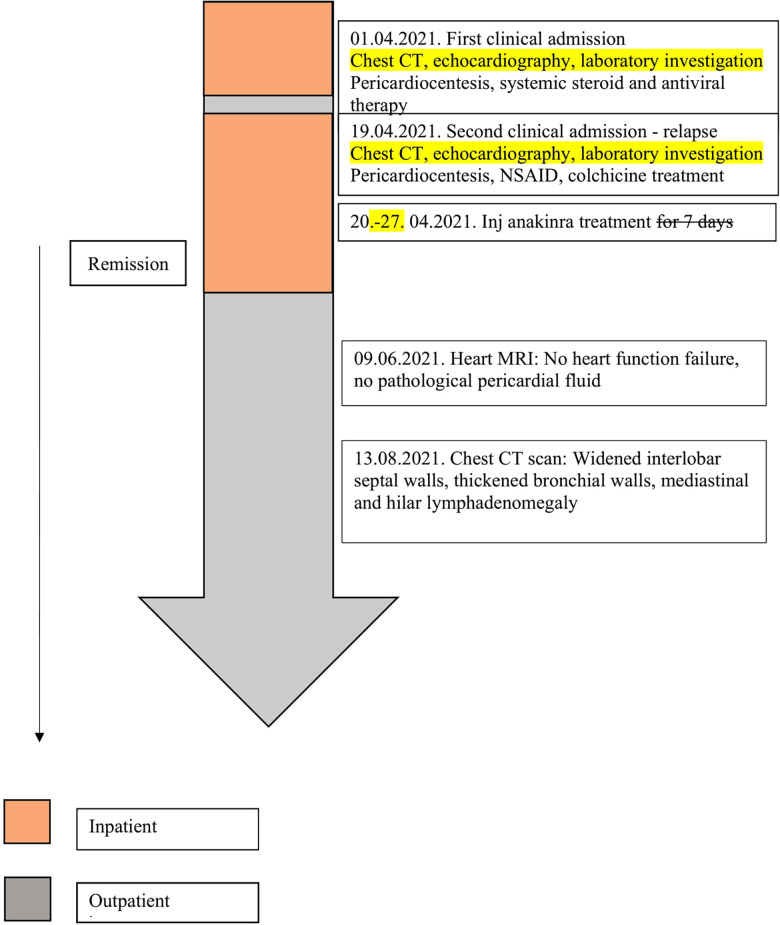
Timeline of care.

Cardiac tamponade is a rare, life-threatening complication of Covid-19 infection. In suspect cases, immediate echocardiography can confirm the diagnosis and help clinicians to intervene in time. In our case, the addition of an interleukine-1 antagonist to the conventional therapy proved to be safe and effective (Figure 3).

## Data Availability

The original contributions presented in the study are included in the article/Supplementary Material, further inquiries can be directed to the corresponding author/s.
